# The Biology and Taxonomy of Head and Body Lice—Implications for Louse-Borne Disease Prevention

**DOI:** 10.1371/journal.ppat.1003724

**Published:** 2013-11-14

**Authors:** Denise L. Bonilla, Lance A. Durden, Marina E. Eremeeva, Gregory A. Dasch

**Affiliations:** 1 Vector-Borne Disease Section, California Department of Public Health, Richmond, California, United States of America; 2 Department of Biology, Georgia Southern University, Statesboro, Georgia, United States of America; 3 Jiann-Ping Hsu College of Public Health, Georgia Southern University, Statesboro, Georgia, United States of America; 4 Rickettsial Zoonoses Branch, Division of Vector-Borne Diseases, Centers for Disease Control and Prevention, Atlanta, Georgia, United States of America; Duke University Medical Center, United States of America

Sucking lice (Phthiraptera: Anoplura) are obligate blood-feeding ectoparasites of placental mammals including humans. Worldwide, more than 550 species have been described and many are specific to a particular host species of mammal [1]. Three taxa uniquely parasitize humans: the head louse, body louse, and crab (pubic) louse. The body louse, in particular, has epidemiological importance because it is a vector of the causative agents of three important human diseases: epidemic typhus, trench fever, and louse-borne relapsing fever. Since the advent of antibiotics and more effective body louse control measures in the 1940s, these diseases have markedly diminished in incidence. However, due to 1) increasing pediculicide resistance in human lice, 2) reemergence of body louse populations in some geographic areas and demographic groups, 3) persistent head louse infestations, and 4) recent detection of body louse-borne pathogens in head lice, lice and louse-borne diseases are an emerging problem worldwide. This mini-review is focused on human body and head lice including their biological relationship to each other and its epidemiological relevance, the status and treatment of human louse-borne diseases, and current approaches to prevention and control of human louse infestations.

## Biological, Genetic, and Taxonomic Relationships between Head Lice and Body Lice

**Figure 1:**
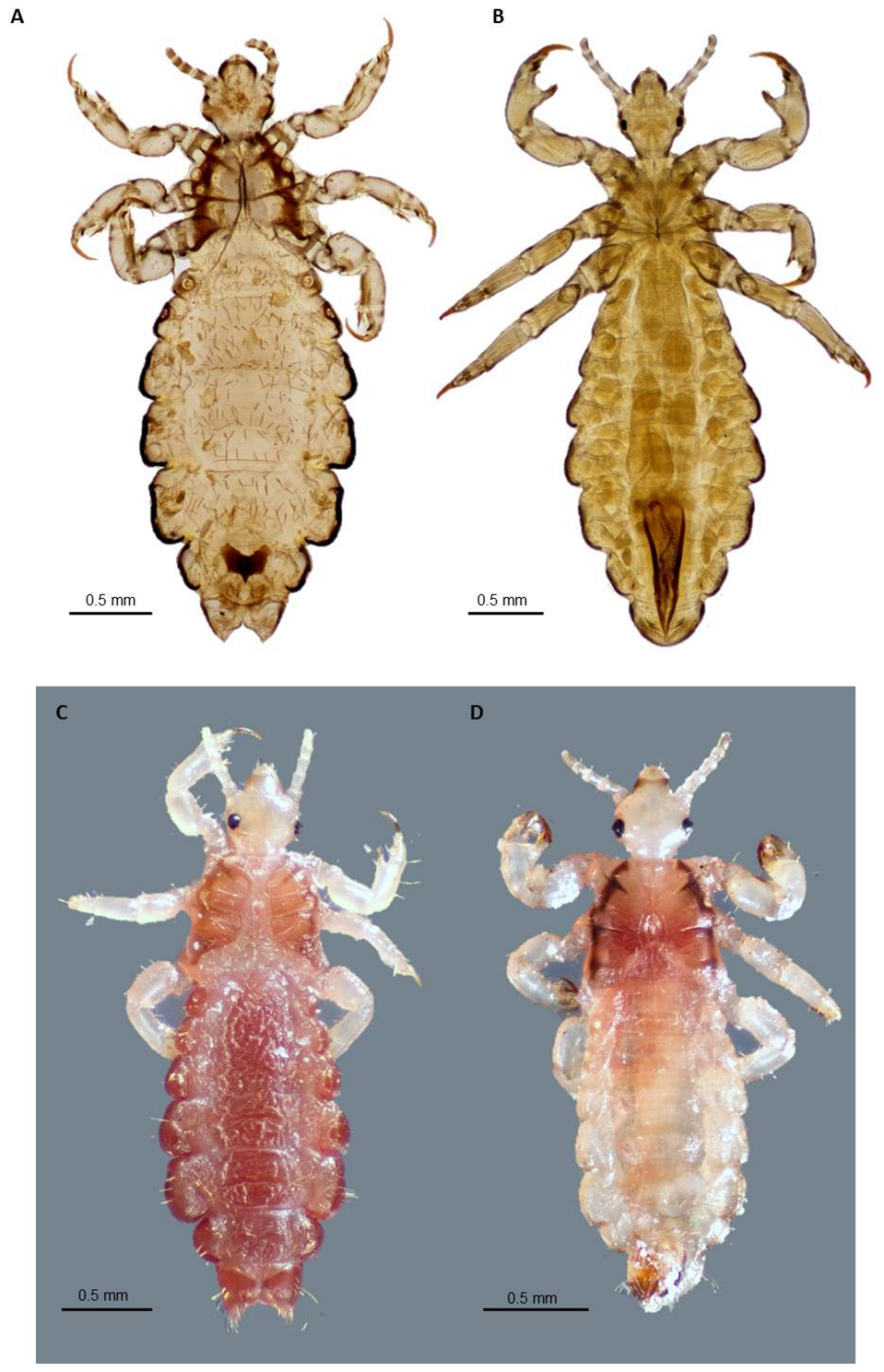
Adult body louse and head lice. A. Ventral view of slide-mounted female head louse; B. Ventral view of slide-mounted male body louse; C. Dorsal view of ethanol-preserved female head louse; D. Dorsal view of ethanol-preserved male head louse. All photographs were taken using a Visionary Digital K2/SC long-distance microscope (Infinity Photo-Optical Company, Boulder, CO), courtesy of Lorenza Beati.

For over a century, scientists have argued about the exact taxonomic and biological relationships between human head lice and body lice and, in particular, whether they represent a single species with two ecotypes or two distinct species [2], [3]. The two-species argument considers the body louse to be *Pediculus humanus* and the head louse to be *Pediculus capitis* (Table 1) (Figure 1A, B, C, D). The single-species argument treats the body louse as *Pediculus humanus humanus* and the head louse as *Pediculus humanus capitis*. Further, although the name *Pediculus humanus corporis* has been used frequently in the medical literature for the body louse, it is an invalid name according to the rules of the International Commission on Zoological Nomenclature. Whether head and body lice represent distinct species, different subspecies (or strains, phylotypes, or ecotypes) inhabiting different habitats, or a single species is more than a taxonomic issue. This is because all well-investigated outbreaks of louse-transmitted diseases in humans, including many that have shaped our history, have involved pathogen transmission by the body louse, not by the head louse [3]. The recent sequencing and annotation of the small 108 Mb genome of *P. humanus humanus*, the chromosome and plasmid of its symbiotic bacterium, “*Candidatus* Riesia pediculicola” [4], [5], and the mitochondria of all three human louse taxa [6] have allowed reevaluation of this argument with potentially important epidemiological ramifications. The sensitivity of lice to sulfamethoxazole-trimethoprim is thought to reflect its lethality for *Riesia*, which lice depend upon for B vitamin synthesis [5], [7]. Differences between head and body lice in the complex developmental interactions that maintain *Riesia* between generations have been described [8], but whether these differences occur in all louse populations is unknown.

**Table 1:** Selected morphological and biological differences between human head and body lice.

CHARACTERISTIC	HEAD LOUSE	BODY LOUSE
Color	Darker	Lighter
Female body length	2.4–3.3 mm	2.4–3.6 mm
Male body length	2.1–2.6 mm	2.3–3.0 mm
Antenna shape	Shorter and wider	Longer and narrower
3^rd^ antennal segment	As long as wide	Slightly longer than wide
Abdominal indentations	Prominent	Not prominent
Apices of paratergal plates	Extending into intersegmental membranes	Not extending into intersegmental membranes
No. eggs laid by females	4–5/day	8–12/day
Oviposition site	Base of head hairs	Clothing fibers esp. along seams
Longevity of adults	Up to 27 days	Up to 60 days
No. bloodmeals	4–10/day	1–5/day
Mitochondrial genetic clades	A, B, C	A

Because of somewhat effective treatment options and increased societal standards for clothing and body hygiene, body lice are currently quite rare in most developed countries [9]. However, they persist or have reemerged in some parts of the world and can also be common in homeless populations in both developed and developing nations [9], [10]. The number of homeless persons has increased significantly in recent decades, and the medical welfare of these people can be difficult to monitor for various reasons [10]. Since homeless persons may not have a change of clothing or be able to adequately delouse their clothes, their garb provides nourishing and unique environments needed for deposition and maintenance of body louse eggs [9], [10]. Explosive increases in populations of body lice have been reported in crowded refugee camps, especially in Africa [9]. Conversely, head lice are common and distributed worldwide with reported infestation prevalences up to 61% [11].

Despite more than 12 years of concerted effort by many investigators to define genetic markers that clearly differentiate head and body louse ecotypes as species or subspecies, this goal has remained elusive [2], [3], [12]–[14]. Most of the data from both mitochondrial and nuclear genes using phylogenetic and population genetic methods fail to clearly separate body and head lice, [2], [12]–[14] indicating that they are conspecific. Although several mitochondrial genes (*cyt*b, COI, and ND4) appear to separate head lice into three clades (A, B, C) and place body lice only in clade A, nuclear genes do not define the same clades, possibly because of modern recombination between different lineages of head lice [2], [12]–[14]. Although these clades exhibit some geographic differences (A is found worldwide, B is found in North America, Central America, Europe, and Australia, and C in Nepal, Ethiopia, and Senegal), it is possible these associations may fail with more extensive sampling of clades B and C [3], [15]. Moreover, in both head and body lice, the 37 mitochondrial genes are located on 18 minicircles, each containing one to three genes, and the intergenic regions are variable in each louse likely as a result of numerous recombination events that make those regions unsuitable for genetic analysis [6]. The ability of head and body lice to interbreed, the common movement of each over both head hair and clothing, and the existence of different color variants of body lice suggest that there has been substantial opportunity for generation of novel genetic variants of *P. humanus* [3], [16], [17]. Indeed, using more robust microsatellite and multispacer typing methods, the two studies that attempted to define genetic differences between head and body louse populations coinfesting the same persons in Nepal or France emphasized opposite conclusions regarding the genetic relationships between the two populations [17], [18]. The louse samples in both studies were small but both clearly demonstrated that individual humans as well as different individuals from the same site can have lice with different genotypes. Indeed, the extensive movement of lice over the human body in populations with both head and body pediculosis makes collections from each louse ecotype rather difficult. An expanded use of microsatellites derived from the genome sequence of body lice was used to differentiate lice from 11 sites in four different geographic regions into geographic clusters [19]. The hope that the global movements of people and their lice have not completely obscured the evolution of lice and the origins of human populations was further enhanced by development of a qPCR assay that distinguished head and body lice from 13 countries based on the PHUM540560 gene, which was expressed differently in transcriptome studies of head and body lice [20], [21]. Although head lice cause medical and psychological problems in their own right, the hypothesis that body lice have emerged repeatedly from head louse populations compounds their potential epidemiological importance [2], [14].

## Louse-Borne Pathogens

The incidence of louse-borne diseases has decreased in humans since the widespread availability of effective antibiotics and pediculicides. Louse-borne relapsing/recurrent fever (RF), caused by infection with *Borrelia recurrentis*, has persisted especially in parts of Africa, and it has the potential to infect travelers returning to Europe and North America from endemic regions [22]. *Borrelia recurrentis* has been detected recently in 23% of head lice in Ethiopia, but whether head lice serve as a vector is unknown [23]. Although the close genetic relationship between *Borrelia duttonii* and *B. recurrentis* has made their laboratory differentiation by qPCR difficult [24], the speculation that acquisition of *B. duttonii* by body lice could quickly give rise to new strains of *B. recurrentis* is uncertain considering the massive loss of protein coding capacity, plasmids, and plasmid rearrangements of the latter [25], [26].

Some other widespread pathogenic bacteria that can be transmitted to humans by other routes, such as *Salmonella typhi* and *Serratia marcescens*, have been detected in human body lice, and *Acinetobacter baumannii* in both head and body lice with the assumption that lice can probably also transmit these agents to humans [27], [28]. There are also experimental and natural observations that human lice are not refractory to *Yersinia pestis*, the causative agent of plague, and that they may be supplementary vectors of this agent [29].

### Trench Fever

*Bartonella quintana* is a bacterium that causes trench fever in humans. It is transmitted by the body louse and possibly by the head louse [3], [9], [30]. Infected lice excrete *B. quintana* onto the skin while feeding, and the bacteria are either scratched into the skin or rubbed into mucous membranes. Historically, trench fever was described in troops in World War I, and again in World War II, but now it is emerging as a problem in urban homeless populations [3]. *B. quintana* has been documented in the homeless and associated body lice from France, the United States, the Netherlands, Ethiopia, Japan, Russia, and Mexico and in refugees, prisoners, and rural populations in Burundi, Rwanda, Zimbabwe, and Peru [30]. *B. quintana* has been found in head lice from homeless people without concurrent body lice infestation [31] as well as in head lice and body lice of different genotypes in Ethiopia [32]. Humans were thought to be the sole reservoir for *B. quintana*, but recently macaque monkeys and their lice, *Pedicinus obtusus*, have also been implicated [33], [34].

### Epidemic (Louse-Borne) Typhus

*Rickettsia prowazekii* is associated with louse and human populations in parts of Africa, South America, and Asia [3]. There is no current circulation of this agent between body lice and humans evident in developed countries of Europe or the Americas. Outbreaks of primary louse-borne epidemic typhus still occur infrequently in Africa. Only sporadic cases of flying squirrel– and tick-associated cases occur in North America as well as rare cases of recrudescent Brill-Zinsser Disease worldwide. Head lice can transmit *R. prowazekii* under laboratory conditions (to naive rhesus macaques and rabbits), and it has been argued that this louse could also be involved in the transmission or maintenance of this pathogen in nature [35], although it has not been detected yet in head lice in nature. Various populations of head lice infesting school children worldwide have tested negative for *R. prowazekii* and/or *B. quintana* despite the presence of both pathogens in body lice from adults in these areas [30]. This potentially indicates the lack of pathogen transmission in pediatric populations or less than critical burdens of these pathogens in head lice.

## Controlling Head and Body Lice Infestations and Related Diseases

At present, there are no commercial vaccines against louse-borne diseases of humans. Therefore, louse-borne disease suppression has typically involved elimination and control of lice and, secondarily, treatment of infected patients with doxycycline [9], [10], [22]. Single-dose oral administration of doxycycline is most effective in controlling epidemic typhus when permethrin dusting of clothing for louse control is not possible. Body louse infestation is typically associated with poor body and clothing hygiene and crowding, which enables close person-to-person contact that facilitates the spread of lice [10]. However, head louse infestations, especially in developed countries, generally have little to do with hygiene, the socio-economic status, or race of the individual, and most frequently affect children between three and 11 years old [11], [36]–[38].

Body louse infestation is diagnosed by finding eggs and crawling lice in the seams and button holes of clothing, and therefore can be controlled by laundering and heat treatment of clothing and wearing permethrin-impregnated clothing [3], [9], [10], [22], [23]. Head lice are frequently viewed as posing no substantial health risk to infested persons but constitute a social embarrassment to parents and children. However, head lice and microbial factors, which can commonly contribute to persistent manifestations including pruritus, head scratching, anemia, and even more severe symptoms, need further investigation. An astonishingly large number of insecticides (Table 2), herbal remedies, occlusive agents, and head lice repellents have been developed to augment physical (combing, vacuuming, heat) methods of louse and nit removal [36]–[38]. However, while pediculicide resistance to over-the-counter treatments, particularly to permethrin and other pyrthethroid derivatives, as well as to other highly efficacious treatments, which may require a prescription, is thought to be widespread, bioassays are difficult to standardize [39] and the correlation of results of genetic assays to ex vivo assays remains problematic [40], [41]. Ivermectin [42] and spinosad lotions appear to be the most promising new treatments, while new molecular approaches to assessments of resistance [20], [40], [43], [44] are making it easier to survey head louse populations for decreasing responsiveness to specific therapies. The challenges that the biology of lice pose for development of customer-friendly, safe, rapid, and effective chemical treatments for killing both live mobile lice and unhatched live eggs are daunting. Unfortunately, herbal remedies and mechanical means do not have the same requirements for measurement of efficacy. However, creative use of nanoparticle and silicone formulations as well as development of safe and effective means to kill head lice in situ offer some expectation that these difficulties will be surmounted [36]–[38].

**Table 2:** Therapeutic options for the treatment of pediculosis.

Pediculicide	Mode of application (concentration)	Chemical composition	Mechanism of action	Effect on head lice^2^	Effect on body lice	Documented resistance in lice	Documented adverse health effect
DDT, dichlorodiphenyltrichloroethane	Dust (10–50%)	Organochloride	Opening of sodium ion channels in neurons	+/+	+	Yes	Toxic
Lindane	Shampoo (1%)Lotion (0.5%)	Organochloride	Inhibition of γ-aminobutyric acid- gated chloride channel	+/−	+	Yes	Toxic
Synergized, natural pyrethrins	Shampoo (0.33%)	Chrysanthemum extract	Delayed repolarization of voltage-gated sodium channels and paralysis	+/−	+	Yes	Minor
Permethrin, synthetic pyrethrin	Cream (1%)Spray (0.5%)^1^	(+)-3-phenoxybenzyl 3-(2,2-dichlorovinyl)-2,2,-dimethyl-cyclopropancarboxylate	The same as natural pyrethrins	+/+^3^	+	Yes^4^	Minor
Malathion	Lotion (0.5%)	Organophosphate	Irreversible inhibition of acetylcholinesterase	+/+	+	Yes	Minor
Ivermectin	Oral tablets (200 µg/kg)	Macrocyclic lactone	Binding to GABA and glutamate-gated chloride ion channels	+/+	^+^	Yes^5^	None to minimal
	Lotion (0.5%)						
Carbaryl	Shampoo (0.5%)	Carbamate	Irreversible inhibition of acetylcholinesterase	+/+	+	Yes	Moderate to very toxic
Spinosad (natroba)	Cream (0.9%)	Mixture of tetracyclic macrolides, spinosyn A and spinosyn D	Overstimulates nerve cells by acting like acetylcholine	+/+	+	no	Minor
Benzyl alcohol lotion	Lotion (5%)	Aromatic alcohol	Asphyxiates lice	+/−	ND	no	Minimal

1 Permethrin is also used for impregnation of clothing for its arthropod repellent properties and as an agricultural pesticide.

2 Indicate information reported about pediculicidal (numerator) and ovicidal (denominator) activity of the insecticide. According to the report of the American Academy of Pediatrics, none of the currently available pediculicides is 100% ovicidal [38].

3 Partial ovicidal activity of permethrin has been reported [41].

4 At baseline, 38% of body lice collected from homeless were resistant to permethrin [43].

5 Possibility of occurrence of ivermectin resistance in body lice has been demonstrated in laboratory settings [44].

## Concluding Remarks

In the 21^st^ century, the prevalence of human louse infestation is still very high worldwide. New molecular tools have been developed and applied to head and body louse ecotypes and to the bacterial agents they transmit. Surprising and novel insights into the evolution of lice, their bacterial disease agents, and the epidemiology of louse-borne diseases have stimulated a renewal of interest in these arthropods. These discoveries may in turn provide new tools for improved understanding and control of these ancient and highly personal scourges of humans.
